# Biological Markers in Early Multiple Sclerosis: the Paved Way for Radiologically Isolated Syndrome

**DOI:** 10.3389/fimmu.2022.866092

**Published:** 2022-04-27

**Authors:** Manon Rival, Manon Galoppin, Eric Thouvenot

**Affiliations:** ^1^ Department of Neurology, Nîmes University Hospital Center, Univ. Montpellier, Nîmes, France; ^2^ IGF, Univ. Montpellier, CNRS, INSERM, Montpellier, France

**Keywords:** multiple sclerosis (MS), radiologically isolated syndrome (RIS), prognosis, biomarkers, personalized medicine, Kappa free-light chain index (kFLC index), glial fibrillary acidic protein (GFAP), neurofilament-light chain (NfL)

## Abstract

Radiologically Isolated Syndrome (RIS) is characterized by MRI-typical brain lesions fulfilling the 2009 Okuda criteria, detected in patients without clinical conditions suggestive of MS. Half of all RIS patients convert to MS within 10 years. The individual course of the disease, however, is highly variable with 12% of RIS converting directly to progressive MS. Demographic and imaging markers have been associated with the risk of clinical MS in RIS: male sex, younger age, infra-tentorial, and spinal cord lesions on the index scan and gadolinium-enhancing lesions on index or follow-up scans. Although not considered as a distinct MS phenotype, RIS certainly shares common pathological features with early active and progressive MS. In this review, we specifically focus on biological markers that may help refine the risk stratification of clinical MS and disability for early treatment. Intrathecal B-cell activation with cerebrospinal fluid (CSF) oligoclonal bands, elevated kappa free light chains, and cytokine production is specific to MS, whereas neurofilament light chain (NfL) levels reflect disease activity associated with neuroaxonal injury. Specific microRNA profiles have been identified in RIS converters in both CSF and blood. CSF levels of chitinases and glial acidic fibrillary protein (GFAP) reflecting astrogliosis might help predict the evolution of RIS to progressive MS. Innovative genomic, proteomic, and metabolomic approaches have provided several new candidate biomarkers to be explored in RIS. Leveraging data from randomized controlled trials and large prospective RIS cohorts with extended follow-up to identify, as early as possible, biomarkers for predicting greater disease severity would be invaluable for counseling patients, managing treatment, and monitoring.

## 1 Introduction

In 2013, the classical definitions of MS clinical courses were modified to take disease activity and disease progression into account (1). Additionally, a clinically isolated syndrome (CIS), the first attack of typical clinical MS symptoms, was defined as early-stage MS, later becoming relapsing–remitting multiple sclerosis (RRMS) if subsequently clinically active and fulfilling the current MS diagnostic criteria (1). Various signs and symptoms (namely, fatigue, pain, bowel and bladder dysfunction, sleep disturbances, and cognitive impairment) and increased healthcare usage may occur in the latent period between the start of neuropathological lesions and CIS, defining the concept of a prodromal phase of MS (2). However, profiles associating multiple biological and clinical features suggestive of MS should be carefully defined to reach appropriate diagnostic specificity before they can be used as markers to screen for MS in populations at risk, such as the offspring of MS patients. In the absence of clinical conditions suggestive of MS, only MRI lesions that fulfill the 2005 dissemination in space criteria (the so-called Okuda criteria) have shown enough specificity for the risk of clinical conversion during follow-up and therefore reached a consensus for the definition of radiologically isolated syndrome (RIS) (3, 4). With this definition, one-third of RIS patients experience their first clinical event, typical of RRMS, after 5 years, while another third show new brain lesions on follow-up scans (5). A long-term retrospective multinational study showed that more than 50% of RIS subjects converted to MS within 10 years, with 11.7% meeting the criteria for primary progressive MS (PPMS) (6).

Predicting the evolution of RIS is of utmost importance for adapting follow-up and therapeutic strategies for effective, personalized care. In large cohorts, male sex and younger age have been identified as baseline predictors of clinical conversion (5–8). Validated MRI prognostic biomarkers are infra-tentorial (IT) and spinal cord (SC) lesions on the index scan and the presence of gadolinium-enhanced (Gd+) lesions on index or follow-up scans (5–8). Recently, studies have shown that the presence of white matter lesions with a central vein sign (CVS) or a paramagnetic rim sign in RIS patients is associated with the presence of SC lesions, suggesting their potential for predicting RIS evolution (9, 10). Optic nerve demyelination identified by visual evoked potentials (VEP), thinning of the peripapillary retinal nerve fiber layer (pRNFL) and the common ganglion cell and inner plexiform layer (GCIP) at baseline and during follow-up on optical coherence tomography (OCT) has also been correlated with a higher risk of clinical conversion (8, 11).

**Figure 1:**
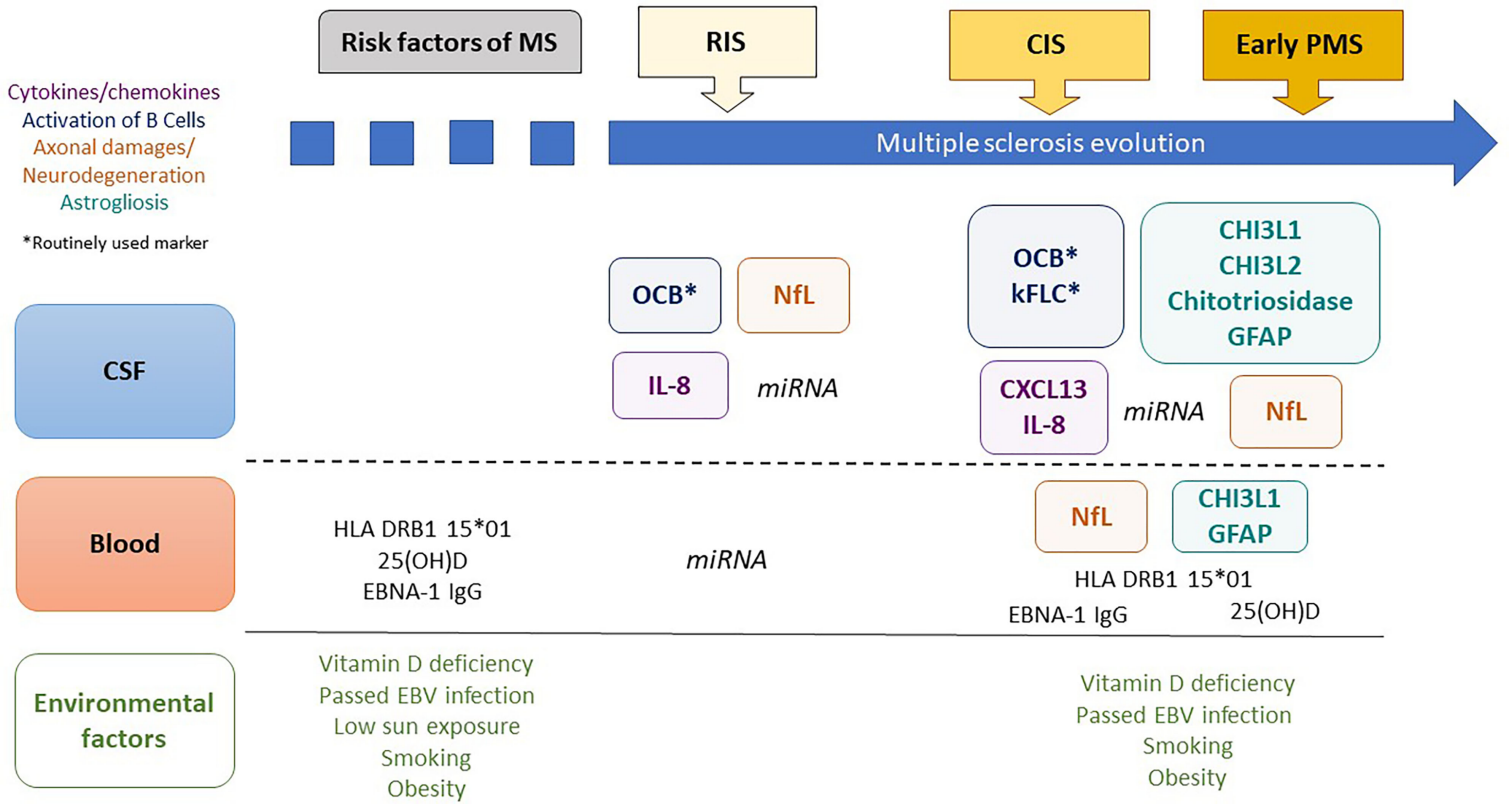
Biological markers predictive of clinical evolution in early multiple sclerosis. MS, multiple sclerosis; CSF, cerebrospinal fluid; RIS, radiologically isolated syndrome; CIS, clinically isolated syndrome; PMS, progressive MS; 25(OH)D, 25-hydroxy vitamin D; EBNA1-IgG, Epstein–Barr Virus-encoded nuclear antigen 1 specific immunoglobulin G; OCBs, oligoclonal bands; CHI3L1, chitinase 3-like protein 1; CHI3L2, chitinase 3-like protein 2; GFAP, glial fibrillary acidic protein; NFL, neurofilament-light chain; kFLC, kappa Free Light Chains; miRNA, microRNA.

Although RIS is not considered a distinct MS phenotype due to the absence of MS symptoms (12), it certainly shares common pathological features with CIS and early progressive MS, encompassing several biological characteristics and markers, forming a set of putative biological markers for the prognosis of RIS (13–15). Except for oligoclonal bands (OCBs) from cerebrospinal fluid (CSF), for 40 years now, have been considered as a biomarker for MS (12), biological markers for early MS remain largely unexplored in RIS. There is a need to identify biomarkers for early MS that may help refine the risk stratification for clinical MS and disability for early treatment. Exploring the pathophysiological pathways for MS involving risk factors for MS, immune system dysfunction, neuroaxonal injury and degeneration, and glial activation in RIS might improve our understanding of this complex disease (16). Additionally, biomarkers for RIS might reveal early pathological features of MS that were unidentified in the later stages and may constitute future therapeutic targets to slow the disease in its pre-symptomatic phase. In this review, we focus on published biological markers predictive of disease activity and progression at the earliest stages of MS, as depicted in **Figure 1**, and discuss their potential interest in RIS subjects.

## 2 Influence of Risk Factors for MS on the Evolution of RIS

In the relatives of MS patients, the risk of MS is much greater and correlates with the degree of kinship, origin, and sex, partly due to several genetic risk factors for MS, especially human leukocyte antigen (HLA) genes (16). Accordingly, there is a higher incidence of RIS in healthy relatives of patients with MS compared to people with healthy relatives (17). HLA-DRB1∗1501 is the main allele responsible for the genetic risk of MS in patients with European ancestry (18). It has also been associated with the risk of clinical events in CIS patients (19), but not in RIS patients (20). Although they are not routinely determined in MS and RIS, analysis of genetic variants associated with MS might still have a minor interest in clinical care.

Low sun exposure, poor vitamin D intake, and low 25-hydroxy vitamin D (25(OH)D) levels in serum, smoking, obesity, and a history of Epstein–Barr virus (EBV) infection are all environmental risk factors for MS (21). Immunoglobulins against EBV-encoded nuclear antigens (EBNA-1,2,3,4,6-IgG) are associated with the risk of developing MS (22). Most of these have also been linked to disease severity (25(OH)D, EBNA1-IgG, obesity, and smoking) (23, 24). Smoking, especially in healthy relatives of patients with MS, is associated with the presence of white matter (WM) signal abnormalities, whereas obesity is related to the presence of ≥9 WM signal abnormalities and fulfillment of the Swanton criteria (17). Lower 25(OH)D levels were associated with the risk of clinical events in a large cohort of CIS patients in univariate analysis, but EBNA1-IgG and smoking status as defined by cotinine levels (>14 ng/ml) were not (25). In a small RIS cohort, there was no difference in 25(OH)D levels in the serum of converters or non-converters (20). The predictive value of 25(OH)D deficiency should be investigated further, as the relatively minor clinical impact of vitamin D therapy in MS may be enhanced if started before disease onset (26, 27).

## 3 Prognostic Biomarker Candidates for RIS

### 3.1 CSF B Cell Lineage and Biomarkers

#### 3.1.1 CSF B Cells

B cells are a key component of acute and chronic inflammatory activity in MS (28), with specific activated clones promoting cytokine production, antigen presentation, differentiation into plasma cells, T cell activation, and CNS invasion by immune cells (29). Inflammatory aggregates of B cells in the subarachnoid spaces were associated with a worse evolution of the disease (30). In analyzing different B-cell subsets (transitional, mature naive, marginal zone, switched memory B cells, IgM-only, IgD-only B cells, and plasmablasts), Guerrier et al. observed that double-negative IgD2/CD272 B cells increased in CIS patients (31). Analysis of the different subsets of T and B cells in RIS could bring new insights into the mechanisms of MS and serve as biomarkers.

#### 3.1.2 Immunoglobulin G and M Intrathecal Synthesis

Clonally expanded B and plasma cells in the CNS locally produce clonal IgGs, leading to CSF restricted oligoclonal bands (OCBs). The presence of OCBs was the first established biological marker for the diagnosis of MS (29) and predicts CIS conversion to clinically definite MS (29). Moreover, RRMS or CIS patients with intrathecal IgG synthesis had a higher risk of and shorter time-to-EDSS worsening over a 4-year follow-up period (32).

In RIS, the presence of OCBs is predictive of clinical conversion in adults (33) and children (34, 35) (**Table 1**), although the presence of OCBs is not correlated with the conversion time in adults (33). Conversely, the IgG index has not shown an independent prognostic value (8, 20). In large cohorts, abnormal CSF, defined as the presence of ≥2 OCBs and/or an IgG index >0.7, revealed a relevant predictive value for disease activity (5, 6, 8). It was also an independent predictor of clinical conversion at 10 years in a multivariate analysis compared to MRI and epidemiological data (6) but not in shorter term studies (5) (**Table 1**). Interestingly, OCBs have been accurately detected in tears and could be used as a minimally-invasive diagnostic tool for RIS if further confirmed in independent cohorts (38).

**Table 1:** Prognostic value of oligoclonal bands and/or IgG index in cerebrospinal fluid in patients with radiologically isolated syndrome.

	Study	Patient characteristics			End-point	Statistical test	Univariate analysis	Multivariate analysis
		N (W%)	Age* (y)	Follow- up* (y)				
Abnormal CSF	Lebrun et al. (8)	70 (75.7)	35.6	5.2	attack	Log-rank test	n.s.	**p = 0.02 ^#^**
	Lebrun et al. (6)	415 (86.5)	37.2	*6.7*	attack or progression	Cox proportional hazards models	HR 2.15 [1.40–3.31] **P <0.001**	HR 1.74 [1.07–2.85] **p = 0.027**
	Okuda et al. (5)	451 (78.4)	37.2	4.4–*2.8*	attack or progression	Cox proportional hazards models	HR 1.78 [1.11–2.87] **p =0.017**	ns
	Thouvenot et al. (36)	71 (76.1)	38.0	*1.3*	attack	Cox proportional hazards models	HR 2.9 [0.83–10.2] p = 0.097	HR 2.22 [0.57–8.59] p = 0.249
	Lebrun et al. (7)	354 (74.6)	38.6	3.8	attack or progression	Cox proportional hazards models	HR 1.26 [0.51–3.09] p = 0.61	–
Oligoclonal Bands	Matute-Blanch et al. (33)	75 (73.3)	36.6	2.8	attack	Cox proportional hazards models	HR 10.31 [1.37–76.61] **p = 0.024**	HR 14.70 [1.80–120.15] **p = 0.012**
	Makhani et al. (34)	38 (71.1)	*15.4*	4.8–*2.5*	attack	Cox proportional hazards models	not shown	HR 10.9 [1.4–86.2] **p = 0.020**
	Makhani et al. (35)	61 (68.9)	*15.0*	4.2–*2.4*	attack	Cox proportional hazards models	HR 4.1 [1.1–14.4] **p = 0.03**	HR 3.0 [1.1–8.5] **p = 0.04**
	Lebrun et al. (8)	70 (75.7)	35.6	5.2	attack	Fisher’s exact test	p = 0.69	NA
	Rossi et al. (37)	18 (50)	29.7	2	attack	Multivariate logistic regression model	not shown	OR 4.45 [0.12–154.07] p **=** 0.400
	Munoz et al. (20)	15 (73.3)	*38*	*6.5*	attack or progression	Fisher’s exact test	p = 0.200	NA
IgG index	Lebrun et al. (8)	70 (75.7)	35.6	5.2	attack	Fisher’s exact test	p = 0.26	NA
	Munoz et al. (20)	15 (73.3)	*38*	*6.5*	attack or progression	Mann–Whitney U test	p = 0.127	NA

Abnormal CSF was defined as IgG index positive (>0.7) and/or the presence of OCBs (≥2). All adult patients fulfilled Okuda’s criteria, children RIS-Ped criteria. *Mean or Median value in years. ^#^significant only among patients with ≥9 T2 lesions on MRI. P-values <0.005 are in bold [95% confidence interval]. N, total number of patients included; W%, percentage of women; HR, hazard ratio; OR, odds ratio; n.s., not significant.

Intrathecal synthesis of IgM has been associated with higher disease activity and shorter progression toward disability compared with abnormal CSF in RRMS patients and an active inflammatory disease phenotype in PPMS patients, but its prognostic value has not been studied for RIS (39, 40).

#### 3.1.3 Kappa-Free Light Chains

Kappa free light chains (kFLC) measured by nephelometry (41) reflect the quantitative intrathecal immunoglobulin synthesis with better accuracy than OCBs and IgG index for MS diagnosis (42) and for predicting clinical conversion in CIS (43), suggesting that it could represent a good candidate biomarker for RIS prognosis. However, studies evaluating small numbers of pooled RIS and CIS patients provide divergent results, and sound investigations of kFLC in RIS are needed (44, 45).

#### 3.1.4 B Cell Cytokines and Chemokines

CXCL13 is a pro-inflammatory chemokine involved mainly in the migration of B cells, a critical stage in the pathology of MS (46). CXCL13 levels assessed in CSF by ELISA have been associated with the conversion of CIS to MS, a higher relapse rate and accumulation of disability (47–49). In only one study of a few RIS patients (n = 4), the CXCL13 index in RIS showed no difference from healthy controls or other stages of MS (50).

In the study by Guerrier, an imbalance in the cytokine production by circulating B cells, especially the alteration of IL-10 production with a high IL-6/IL-10-producing B-cell ratio, was associated with clinical conversion and its delay in a mixed cohort of CIS and RIS patients (31). Concentrations of B cell-related factors, notably CD27, FCRL2, CXCL10, and CXCL13, increase in MS CSF, especially in the early stages of the disease (51). Further studies must confirm B-cell phenotyping as a valuable prognostic biomarker.

### 3.2 Other Inflammatory Biomarkers

#### 3.2.1 Soluble CD27

A soluble form of CD27 (sCD27) is released by activated T cells and co-stimulates B and T cell activation and proliferation in autoimmune diseases like MS (52–54). High sCD27 levels in the CSF of CIS patients have been associated with a 5.5 times higher annual relapse rate (53) and the CSF sCD27/T-cell ratio increases in progressive MS (55). However, serum sCD27 levels do not discriminate between MS patients and healthy individuals (54).

#### 3.2.2 Interleukin-8

Interleukin-8 (IL-8) is a pro-inflammatory chemokine produced by astrocytes and microglia in response to active intrathecal inflammation (56). It activates monocytes and neutrophils (37) and binds to oligodendrocytes and hypertrophic astrocytes in MS (57). Elevated CSF IL-8 levels are predictive of MS conversion following a CIS (37). In a small group of 18 RIS patients, a high level of CSF IL-8 was an independent predictor of clinical conversion (37), making IL-8 a candidate for RIS prognosis to be further validated.

#### 3.2.3 Interleukin 17A

Studies on experimental autoimmune encephalomyelitis, an animal model of MS, highlighted the role of Th17 lymphocytes, characterized by interleukin 17A (IL-17A) secretion, as strong inducers of pro-inflammatory responses (58). In a large cohort of 1,327 MS spectrum patients (RIS-CIS-RRMS), IL-17A levels were higher than in healthy controls in CSF but not in serum (59). Serum and CSF IL-17A did not discriminate between MS subtypes and did not demonstrate any prognostic value in 35 RIS patients (59).

### 3.3 Markers of Neuroaxonal Damage and Glial Activation

#### 3.3.1 Neurofilaments

Neurofilaments encompass a family of 5 intermediate filaments (heavy, medium, light chains (NfL), a-internexin, and peripherin) involved in axonal growth and stability as well as mitochondrial and synaptic functions in central and peripheral neurons (60). Neurofilaments can be released into the interstitial fluid from injured neurons, either due to the loss of neuronal membrane integrity or to active secretion related to axonal damage or neurodegeneration. According to other brain protein clearance, degraded neurofilaments may be absorbed from interstitial fluid into lymphatic vessels or directly absorbed by the blood vessels *via* perivascular drainage along the basement membranes of capillaries (61). Different levels of blood–brain barrier leakage induced by inflammation probably modify the kinetics of the neurofilament-light chain, circulating between the brain and blood compartments and its final blood concentration (60). NfL in CSF (cNfL) has been associated with clinical activity in CIS patients (62). cNFL can tell RIS apart from RRMS and PPMS, but not from early-stage CIS or healthy controls (63). Among 75 RIS patients, high cNfL measured by ELISA (Uman-Diagnostics; Umeå, Sweden) has been associated with an increased risk of conversion to CIS or to RRMS (CIS was based on the 2010 McDonald criteria in this study) (33).

Recently, ultrasensitive technologies such as the single molecule array (Simoa™) and the microfluidic platform (Simple Plex™ Ella) have been developed, allowing for the accurate determination of NfL levels in serum (sNfL) and highly correlated cNfL levels (64, 65). Using Simoa™, sNfL levels have been associated with disease activity, treatment response, and long-term outcomes at different stages of MS (66, 67) and identified as an independent predictor of relapse in newly-diagnosed MS and CIS patients (68, 69). The prognostic value of sNfL has not been investigated in RIS subjects. However, in a large epidemiological study among US military personnel, it was significantly higher among people who developed MS within 6 years (70). sNfL might provide a potentially less invasive option for assessing RIS prognosis when a lumbar puncture cannot be performed.

#### 3.3.2 Glial Fibrillary Acidic Protein

Glial Fibrillary Acidic Protein (GFAP) measurement has recently been implemented with NfL in multiplex kits (2-PLEX B and 4-PLEX A) by Quanterix^®^, making it possible to investigate astrocytic activation along with neuroaxonal damage in serum samples. GFAP is one of the major intermediate filament proteins expressed in astrocytes. CSF GFAP levels correlate with different subtypes of MS, reflecting different degrees of damage to astrocytes and may represent a useful marker of disease progression (71). CSF and serum GFAP (sGFAP) levels are correlated with MS patients (72). sGFAP has been associated with a higher Expanded Disability Status Scale (EDSS) score, older age, longer disease duration, progressive disease course, and MRI pathology (73, 74). The positive correlation between sGFAP and the clinical severity of the disease may highlight a particular role of astrocytes in progressive MS and mark the potential of sGFAP as a marker of disease severity (73). In RIS, the prognostic value of sGFAP as a minimally invasive biomarker of conversion to PPMS should be evaluated.

#### 3.3.3 Chitinase 3-Like protein 1

Chitinase 3-like protein 1 (CHI3L1, also known as YKL-40) is a protein of the chitin family mainly released in the CNS by activated astrocytes (75), microglia, and macrophages (76) in response to acute and chronic inflammation. It has been described as inhibiting oxidant-induced injury, increasing Th2 immunity, and regulating apoptosis (77). CSF CHI3L1 levels (cCHI3L1) measured by ELISA predict conversion from CIS to clinically definite MS and development of disability (75, 78). Indeed, cCHI3L1may reflect non-lymphocytic low-grade inflammation leading to active neurodegeneration (79), explaining its association with neurological disability quantified by EDSS in PPMS (80). However, all studies consistently show the absence of prognostic value of cCHI3L1 in RIS (20, 33, 36), suggesting that astrocytic and microglial activation is too scarce at the pre-symptomatic stage of MS. However, chitotriosidase and chitinase 3-like protein 2 (CHI3L2), two other members of the chitin family with similar properties, also need to be evaluated (75, 81, 82).

Although at a much lower concentration than in the CSF, ELISA made it possible to quantify serum CHI3L1 (sCHI3L1) levels, which are also associated with the risk of conversion to RRMS in CIS patients (75). Additionally, sCHI3L1 is higher in PMS patients than in RRMS patients and correlates with disability as determined by EDSS in PMS patients (83). However, the prognostic value of sCHI3L1 for the conversion to CIS or to PMS in RIS patients has not been assessed.

Altogether, NfL, likely associated with acute neuroaxonal injury, might have an interesting predictive value in the early stages of MS for disease activity, whereas GFAP and sCHI3L1 seem rather to be associated with glial activation, and could be of interest for predicting conversions to progressive MS. Their association in a CSF or serum “glia score” (GFAP*CHI3L1/NfL) better discriminates RRMS vs. PPMS than each biomarker alone, CSF being more accurate than serum (AUC 0.80 vs. 0.68, respectively) (83).

### 3.4 Innovative Genomic, Proteomic, and Metabolomic Approaches

#### 3.4.1 MicroRNA

MicroRNA (miRNA) is an extremely stable class of non-coding single-stranded RNA with post-transcriptional regulatory functions (84) that can be detected in peripheral blood or CSF. Some serum and CSF miRNA profiles have been associated with MS (84, 85), while others predict clinical evolution in CIS patients (86). In 15 RIS patients, miRNA specific profiles in CSF (miR-144-3p, miR-448, and miR-653-3p) and in plasma (miR-142-3p, miR-338-3p, miR-363-3p, miR-374b-5p, miR-424-5p, and miR-483-3p) have been associated with the risk of conversion after 5 years of follow-up (20) and require further validation.

#### 3.4.2 Mass Cytometry

Mass cytometry (CyTOF) can help decipher immune cell phenotypes. In CSF from early MS patients, a B-cell population expressing CD49d, CD69, CD27, CXCR3, and HLA-DR could be a strong candidate for an MS-specific cell type (51). In the blood of CIS patients, an increased proportion of both a T-bet-expressing B cell subset and a CD206+ classical monocyte subset has been identified, especially in very active MS patients (disease activity after 6 months of disease modifying therapy or two or more relapses within one year with residual disability and radiological activity) (87).

These approaches provide new insights into the pathophysiology of MS and allow the identification of immunological biomarkers of early MS. Further studies will be required to determine the exact role of new candidate biomarkers and validate their diagnostic and prognostic value in RIS patients.

#### 3.4.3 Proteomics and Metabolomics

In the past few years, technical breakthroughs have made it possible to screen for many molecules as candidate biomarkers through unbiased -omic approaches. SOMAscan™ has identified specific protein profiles in the CSF extracellular vesicles of RRMS patients (88). The Olink inflammation panel has identified CCL11 and CCL20 as plasma biomarkers associated with MS progression and severity (89).

Metabolomics can identify the disturbed pathways involved in signaling and energy supply, providing potential signature profiles for MS diagnosis, stages, and assessment of drug responses, especially involving the alpha-linoleic acid pathway, nucleotide metabolism, amino acid metabolism, tricarboxylic acid cycle, D-ornithine, and D-arginine pathways (90).

The multi-omics-based algorithm based on protein profiling by SOMAScan™ and nuclear magnetic resonance metabolite measures has outperformed the current individual biomarkers for predicting the risk of conversion to clinically definite MS in CIS patients (91), although a reproducible MS-specific metabolome-based signature remains to be identified. Applied to RIS, these approaches could bring new insights into the molecular pathways promoting the disease and more accurately predict individual prognoses.

## 4 Discussion

Prognostic values of several biological factors have been tested in RIS owing to their interest in different subtypes of MS, especially in CIS and early progressive MS.

First, the most studied biomarker in MS and validated MS diagnostic criteria, OCBs, remains the most relevant prognostic biomarker for RIS. Physiologically linked to OCBs and with greater accuracy in other phases of the disease, kFLC might be a good candidate prognostic biomarker for RIS.

Secondly, although unavailable in routine clinical care, data concerning NfL, IL-8, and miRNA profiles in CSF have encouraged us to explore their potential as biomarkers for RIS prognosis (**Figure 1**). Additionally, CHI3L1 and GFAP, reflecting glial activation, need to be explored in CSF as possible biomarkers for early PPMS and disability progression.

Finally, no peripheral biological markers have so far been identified as providing additional prognostic value, except for the miRNA profile. CHI3L1, GFAP, and NfL, accurately measurable in blood, might also constitute potential peripheral biomarkers of disease activity and progression.

Along with candidate biomarkers from current knowledge of early MS and -omics approaches, therapeutic response biomarkers may arise from ongoing randomized controlled trials (RCTs) in RIS subjects [TERIS, NCT03122652 (92) and ARISE, NCT02739542 (93)]. Leveraging samples and data from RIS patients in RCTs and large prospective cohorts with extended follow-up will be necessary to validate these candidate biomarkers for RIS, which predict greater disease severity. Moreover, identifying biological biomarkers obtained from blood samples—far less invasive than a lumbar puncture—should be a priority for future studies.

## Author Contributions

MR wrote the first draft of the manuscript, wrote sections of the manuscript, and contributed to manuscript revision, read, and approved the submitted version. MG contributed to manuscript revision, read, and approved the submitted version. ET contributed to the conception and design of the study, wrote sections of the manuscript, contributed to manuscript revision, read, and approved the submitted version. All authors listed have made a substantial, direct, and intellectual contribution to the work and approved it for publication.

## Conflict of Interest

ET received fees, travelling expenses and research grants from the following pharmaceutical companies: Actelion, Biogen, Genzyme, Merck Serono, Novartis, Roche, Teva pharma.

The remaining authors declare that the research was conducted in the absence of any commercial or financial relationships that could be construed as a potential conflict of interest.

## Publisher’s Note

All claims expressed in this article are solely those of the authors and do not necessarily represent those of their affiliated organizations, or those of the publisher, the editors and the reviewers. Any product that may be evaluated in this article, or claim that may be made by its manufacturer, is not guaranteed or endorsed by the publisher.
